# Editorial: Mitochondrial Remodeling and Dynamic Inter-Organellar Contacts in Cardiovascular Physiopathology

**DOI:** 10.3389/fcell.2021.679725

**Published:** 2021-04-30

**Authors:** Gaetano Santulli, Giovanni Monaco, Valentina Parra, Giampaolo Morciano

**Affiliations:** ^1^Division of Cardiology, Department of Medicine, Albert Einstein College of Medicine, Wilf Family Cardiovascular Research Institute and Einstein Institute for Aging Research, New York, NY, United States; ^2^Department of Molecular Pharmacology, Einstein-Sinai Diabetes Research Center (ES-DRC), Fleischer Institute for Diabetes and Metabolism (FIDAM), Montefiore University Hospital, New York, NY, United States; ^3^International Translational Research and Medical Education Academic Research Unit (ITME), Department of Advanced Biomedical Sciences, “Federico II” University, Naples, Italy; ^4^Center for Innovation and Stimulation of Drug Discovery (CISTIM), Leuven, Belgium; ^5^Laboratory of Molecular and Cellular Signaling, Department of Cellular and Molecular Medicine, KU Leuven, Leuven, Belgium; ^6^Departamento de Bioquímica y Biología Molecular, Facultad de Ciencias Químicas y Farmacéuticas, Universidad de Chile, Santiago, Chile; ^7^Advanced Center of Chronic Diseases (ACCDiS), Facultad de Ciencias Químicas y Farmacéuticas y Facultad de Medicina, Universidad de Chile, Santiago, Chile; ^8^Network for the Study of High-Lethality Cardiopulmonary Diseases (REECPAL), Universidad de Chile, Santiago, Chile; ^9^Department of Medical Sciences, Section of Experimental Medicine, Laboratory for Technologies of Advanced Therapies (LTTA), University of Ferrara, Ferrara, Italy; ^10^Maria Cecilia Hospital, GVM Care and Research, Cotignola, Italy

**Keywords:** cardiovascular disease, mitochondria, sarcoplasmic reticulum, contact sites, signal transduction

Emerging evidence has shown that membranes of many subcellular organelles are dynamic and engage in structural and functional communications, thereby creating new intracellular compartments by either sharing proteins or by owning a distinct pool (Rizzuto et al., 1998; Giorgi et al., 2018).

Membranes from different organelles do not fuse together, but preserve their integrity by approaching not more than a few nanometers (usually 10 nm); this distance is enough to create transient contacts which significantly impact physiological processes (e.g., lipid metabolism, material exchange) (Simmen and Tagaya, 2017; Vance, 2020) and human diseases (van Vliet and Agostinis, 2018; Simoes et al., 2020).

Growing advances in technologies, including cell fractionation (Wieckowski et al., 2009; Montesinos and Area-Gomez, 2020), confocal (Chung et al., 2015; Galmes et al., 2016), and transmission electron microscopy (Csordás et al., 2006), alongside new tools which combine biochemistry and online databases (e.g., Contact-ID) (Kwak et al., 2020), have allowed the study of contact sites in many types of living cells, in order to address new structural, functional, and modulatory properties.

Contact sites in cardiomyocytes, especially those established between sarcoplasmic reticulum (SR), and transverse tubules (TT) of the sarcolemma, and with mitochondrial membranes, are necessary for excitation-contraction coupling (ECC) efficiency (Gambardella et al., 2018) and suitable calcium signaling (Fearnley et al., 2011). The latter sustains cell survival by modulating mitochondrial ATP generation to match cardiac workload and also cell death (Jouaville et al., 1999; Traaseth et al., 2004; Bonora et al., 2019).

Among the intracellular organelles, mitochondria play an essential role in cardiomyocyte bioenergetics, because they constitute 35% of the total cell volume to satisfy the high-energy demand of heart (Elfering et al., 2004; Benard et al., 2007). As such, it is not surprising that mitochondrial dysfunction underlies several defects observed during heart development and differentiation, participating actively in the pathogenesis of a number of cardiovascular diseases (Santulli et al., 2015; Bravo-Sagua et al., 2020). Hence, maintaining a healthy mitochondrial population is an essential homeostatic requirement that the cell retains by controlling multiple checkpoints including a balanced ratio between mitophagy and biogenesis, including mitochondrial fission and fusion (Morciano et al., 2020).

The present collection includes 11 reports subdivided in the following categories: basic mechanisms, human diseases, and therapies.

## Basic Mechanisms

Five out of 11 reports belong to this category and are authored by Rossini and Filadi, Lin et al., Gilkerson et al., Lynch et al., and Piquereau et al.. The authors highlighted the importance of the cytoarchitecture, especially SR-mitochondria contact sites and spatio-temporal mitochondrial remodeling, in some molecular pathways essential for cardiomyocyte function. These include calcium signaling, one of the main players in mitochondrial bioenergetics and cardiac contractility; in this context, organelles and proteins involved in intracellular calcium fluxes have been analyzed both *in vitro* and *in vivo*. Moreover, new insights have been provided about reactive oxygen species (ROS) production, mitochondrial dynamics, and quality control in the adaptation of the heart to multiple stress conditions. Lastly, there is a report highlighting the ability of sex hormones as factors able to influence metabolism via mitochondrial remodeling.

## Diseases

Four manuscripts authored by Gao et al., Salazar-Ramírez et al., Ramaccini et al., and Kumar et al. report compelling evidence of how mitochondrial dysfunction and alterations in organelle communication can impact cellular homeostasis in cardiovascular diseases. Indeed, the rewiring of calcium signaling at SR-mitochondria interface (but also at the sarcolemma), the imbalance in mitophagy, defects in fusion-fission machinery, lipid biosynthesis, ATP and ROS production are analyzed in a wide range of pathologies including dilated cardiomyopathy (DCM), heart failure, ischemia-reperfusion injury, and cardiac arrythmia.

## Therapies

In CVD, the altered mitochondrial remodeling and impaired inter-organellar communications of cardiomyocytes may be amenable to therapeutic interventions, especially considering the dynamic and reversible nature of these interactions (Ferrandi et al., 2013; Sabbah, 2016; Siasos et al., 2018; Kerkhofs et al., 2019). In this sense, the last 2 reports authored by Elorza et al. and Angebault et al. summarize the currently available therapies targeting mitochondrial fitness (e.g., maintaining the correct balance of biogenesis and the control of mitochondrial heteroplasmy to prevent age-related diseases) and report the beneficial effects of metformin in mice affected by Duchenne muscular dystrophy (DMD)-associated cardiomyopathy. In this preclinical model, metformin was able to normalize SR-mitochondria interactions, and restore the function of the electron transport chain (ETC) Complex I and the expression of mitochondrial calcium-handling protein complexes.

## Author Contributions

GS and GMor conceived, wrote, and finalized the Editorial. GMon and VP wrote the Editorial.

## Conflict of Interest

The authors declare that the research was conducted in the absence of any commercial or financial relationships that could be construed as a potential conflict of interest.
